# Spotted fever group rickettsiae and *Anaplasma phagocytophilum* in *Borrelia burgdorferi sensu lato* seropositive individuals with or without Lyme disease: A retrospective analysis

**DOI:** 10.1016/j.nmni.2023.101139

**Published:** 2023-04-24

**Authors:** Leonie Kosak, Norbert Satz, Markus Jutzi, Marinko Dobec, Patricia Schlagenhauf

**Affiliations:** a University of Zürich, Institute for Epidemiology, Biostatistics and Prevention, Zürich, Switzerland; b Praxis Am Paradeplatz, Zürich, Switzerland; c Analytica Medizinische Laboratorien AG, Zürich, Switzerland; d University of Zürich, Institute for Epidemiology, Biostatistics and Prevention, WHO Collaborating Centre for Travellers' Health, Department of Global and Public Health, MilMedBiol Competence Centre, Zürich, Switzerland

**Keywords:** SFG rickettsiae, Co-infection, *Anaplasma phagocytophilum*, Lyme disease, Tick bite, Borrelia, Switzerland, Acrodermatitis atrophicans chronica

## Abstract

**Background:**

The *Ixodes ricinus* tick is the main vector of *Borrelia burgdorferi* and tick-borne encephalitis virus in Switzerland*.* Spotted fever group Rickettsiae (SFG) and *Anaplasma phagocytophilum* have been detected in Swiss ticks, however, information about the extent and clinical presentation of these infections in humans is scant.

**Methods:**

Indirect fluorescent antibody tests for SFG rickettsiae and *Anaplasma phagocytophilum* were performed on serum samples of 121 *Borrelia burgdorferi* seropositive patients with and without Lyme disease and 43 negative controls.

**Results:**

Out of 121 *Borrelia burgdorferi* seropositive individuals, 65 (53.7%) were seropositive for IgG and 15 (12.4%) for IgM antibodies to SFG rickettsiae. IgM antibodies were detected more frequently in early-than in late-stage of Lyme disease (12 out of 51 and 2 out of 49; respectively; p ​= ​0.0078). Significantly higher IgG antibody titers against SFG rickettsiae were found in patients with late-stage compared to patients with early-stage Lyme disease (mean titer 1:261 and 1:129, respectively; p ​= ​0.038). This difference was even more pronounced in patients with acrodermatitis chronica atrophicans compared to patients with early stage of Lyme disease (mean titer 1:337 and 1:129, respectively; p ​= ​0.009).

In patients presenting with fatigue, headache and myalgia, the prevalence of IgG antibodies against SFG rickettsiae was significantly higher (7 out of 11; 63.6%) than in *Borrelia burgdorferi* seropositive individuals without clinical illness (1 out of 10; 10%; p ​= ​0.024). IgG antibodies to *Anaplasma phagocytophilum* were detected in 12 out of 121 individuals (9.9%), no IgM antibodies were found.

**Conclusion:**

Infections with SFG rickettsiae and *Anaplasma phagocytophilum* are underdiagnosed and should be ruled out after a tick bite. Further studies are needed to elucidate the possible causative role of SFG rickettsiae for myalgia, headache and long-lasting fatigue after a tick bite and to determine the necessity for an antibiotic treatment.

## 1 Introduction

*Ixodes ricinus (I. ricinus)* is the most common and best-known species of shield ticks (Ixodidae) in Switzerland and Central Europe [1]. It is the vector for *Borrelia burgdorferi sensu l*ato *(B. burgdorferi)* and the tick-borne encephalitis virus (TBEV) that cause Lyme disease and tick-borne encephalitis (TBE) respectively [2,3]. For these tickborne diseases an increasing incidence has been observed over the past several years [[4], [5], [6]].

Additionally, other microorganisms, including *Rickettsia helvetica (R. helvetica), R. monacensis, R. slovaca* and *Anaplasma phagocytophilum (A. phagocytophilum)* have been detected in *I. ricinus* ticks [[7], [8], [9]]. During a tick bite, these microorganisms can be transmitted to humans and sometimes lead to infection [10].

Tick-borne rickettsioses are caused by spotted fever group (SFG) rickettsiae. They can be found across the globe and can cause infections of varying clinical severity, with symptom onset usually within four to ten days after a tick bite. The most common symptoms are fever, muscle pain, headache, rash and eschar [11,12]. Diagnosis is based on detection of IgM and IgG antibodies against SFG rickettsiae. The sensitivity of IgG antibody detection 5–9 days after disease onset is 46% and increases to approximately 100% after four weeks [13].

Currently, at least 15 different *Rickettsia species* have been shown to be associated with SFG rickettsiosis in humans. The three best known members are R*. rickettsii*, the causative agent of Rocky Mountain spotted fever, *R. conorii*, the causative agent of Mediterranean spotted fever and *R. africae,* the causative agent of African tick bite fever [14,15].

Of the two *Rickettsia* spp. endemic in Switzerland, *R. helvetica* is the most widespread and is also one of the most encountered tick-borne rickettsiae in *I. ricinus* throughout Europe [10]. In forest areas of the Canton of Zurich, *R. helvetica* DNA was detected in 30.0% of *I. ricinus* ticks [9]. Although *R. helvetica* and *R. monacensis* have been isolated and cultured from *I. ricinus* and human serum, their pathogenic potential has not been fully elucidated. So far, sporadically symptoms ranging from unexplained fever, fatigue, myalgia, arthralgia to perimyocarditis and meningitis have been described [11,[16], [17], [18]].

The infection rate of *I. ricinus* ticks in forest areas of Zürich for *A. phagocytophilum* was 3.0% [9]. *A. phagocytophilum* is the causative agent of the bacterial infectious disease human granulocytic anaplasmosis. After an incubation period of 5–14 days the infection can present with high fever, flu-like symptoms, headache, limb, muscle and joint pain. However, this infection can range from subclinical symptoms to potentially life-threatening, multiorgan failure [19].

These different microorganisms harboured by *I. ricinus* can be transmitted through a tick bite. Several studies have shown that co-infections are more widespread than originally thought [[20], [21], [22]].

This retrospective serological study aimed to investigate the seroprevalence of SFG rickettsiae and *A. phagocytophilum* in persons who experienced a tick bite. *B. burgdorferi* seropositivity was used as a surrogate marker for a tick-bite. Subsequently, disease symptoms were analysed in patients with monoinfections (*B. burgdorferi*) and co-infections.

## 2 Material and methods

### 2.1 Study design, participants, and selection criteria

We conducted a retrospective study including antibody detection in archived serum samples and patient chart review. Ethics approval was received by the Ethics Commission of the Canton of Zurich (Basec-Nr. 2021-01934). Informed consent was obtained from all control subjects and patients for study participation including testing of their serum samples and patient chart review. Patient serum samples were collected during spring, summer and fall 2020–2022. Serum samples of the control group were collected during April 2021. All sera were stored at −20 ​°C until use. Subjects in the patient and control groups live in the same area in Switzerland, mainly in the canton of Zurich, and therefore have an equal potential for exposure to SFG rickettsiae and *A. phagocytophilum.*

All patients who had previously been tested positive for antibodies against *B. burgdorferi* upon request by the “Praxis am Paradeplatz” and for whom archived serum samples were available as per December 9, 2021, were invited by letter to participate in our study. Written informed consent was received from 121 of these 204 patients. With a letter from December 6, all 240 employees of Analytica Medizinische Laboratorien AG were invited to be part of the control group. Out of 77 volunteers, 43 met the inclusion criteria (negative for IgG and IgM antibodies against *B. burgdorferi* in a current serum ample, no previous clinical evidence of infection with *B. burgdorferi*, no history of tick bite or residence in SFG rickettsial endemic areas so far).

Of the 121 ​*B. burgdorferi* seropositive individuals, 100 showed clinical manifestation of Lyme disease according to the EUCALB-criteria (European Concerted Action against Lyme Borreliosis; group 1), and 21 presented with other clinical symptoms non-specific for Lyme disease or without clinical disease (group 2). The patient group consisted of 57 men (age range 14–83, median age 52.9 years) and 64 women (age range 7–86, median age 56.5 years).

Within group 1, 51 patients had an early-stage Lyme disease (50 patients with erythema migrans and one patient with Borrelia lymphocytoma), while 49 patients presented with late-stage symptoms (11 patients had Lyme arthritis, 11 neuroborreliosis and 27 had acrodermatitis chronica athrophicans (ACA).

According to their clinical presentation, patients of group 2 were further divided into *B. burgdorferi* seropositive patients presenting with myalgia, headache, and fatigue (n ​= ​11, group 2a) and *B. burgdorferi* seropositive individuals without clinical disease (n ​= ​10, group 2b).

The control group comprised 43 volunteers, 12 men and 31 women (age range 28–62, median age 46.9 years).

### 2.2 Antibody detection

For the detection of IgG and IgM antibodies against *B. burgdorferi* a chemiluminescence immunoassay (CLIA) as a screening test was used. (Borrelia IgM-/IgG LIAISON XL, DiaSorin, Saluggia, Italy). In case of positive or borderline result, a confirmatory immunoblot test was performed (Borrelia, ViraStripe IgG/IgM Test, Viramed Biotech AG, Planegg, Germany). All tests were performed and interpreted according to the manufacturer's instructions.

IgM and IgG antibodies to SFG rickettsiae and *A. phagocytophilum* were detected by indirect immunofluorescence assay according to the manufacturer's instructions (*Rickettsia rickettsii* IFA IgG/IgM and *Anaplasma phagocytophilum* IFA IgG/IgM, Focus Diagnostics, Cypress, California, USA). For the detection of antibodies to *A. phagocytophilum* a species-specific antigen was applied. On the other hand, a lipopolysaccharide (LPS) group-specific *R. ricketsii* antigen served as a cross-reacting component, allowing detection of antibodies to SFG rickettsiae without species discrimination. All tests are CE-marked in-vitro diagnostics.

### 2.3 Statistical analysis

Significant differences between groups were determined by the two-tailed Fisher's exact test and the confidence interval of proportion was calculated (GraphPad, Software, La Jolla, California, USA). The Mann-Whitney *U* test was used to compare antibody titers to SFG rickettsiae between patients in early and late stages of Lyme disease (GraphPad Software, La Jolla, California, USA). P-values <0.05 were considered statistically significant.

## 3 Results

### 3.1 Serology and clinical presentation

**Fig. 1:**
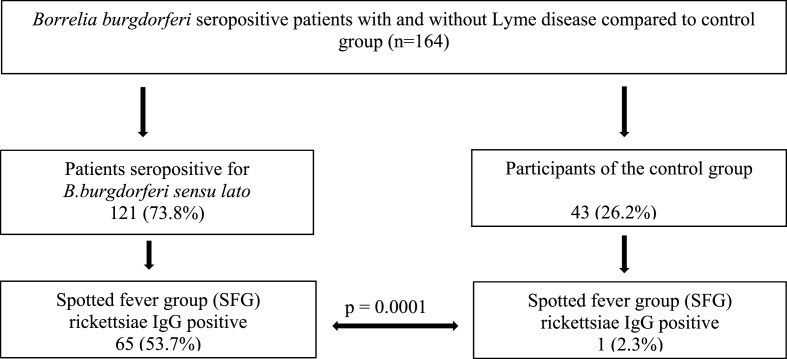
Seroprevalence of spotted fever group (SFG) rickettsiae in *Borrelia burgdorferi* seropositive patients with and without Lyme disease compared to control group.

A statistically significant difference in the seroprevalence of IgG antibodies against SFG rickettsiae was observed between *B. burgdorferi* seropositive patients (65 out of 121; 53.7%; 95% CI, 0.4486 to 0.6235) and *B. burgdorferi* seronegative controls (1 out of 43; 2.3%; 95% CI, <0.0001 to 0.1316; p ​= ​0.0001; Fig. 1).

IgG antibodies against SFG rickettsiae were detected in 57 out of 100 (57%; 95% CI, 0.4721 to 0.6627) and IgM in 14 out of 100 patients (14%; 95% CI, 0.084 to 0.2226) and this difference is significant (p ​< ​0.0001). IgG and IgM antibodies against SFG rickettsiae were simultaneously detected in 8 out of 100 patients (8%; 95% CI, 0.039 to 0.1521) with Lyme disease (group 1).

**Fig. 2:**
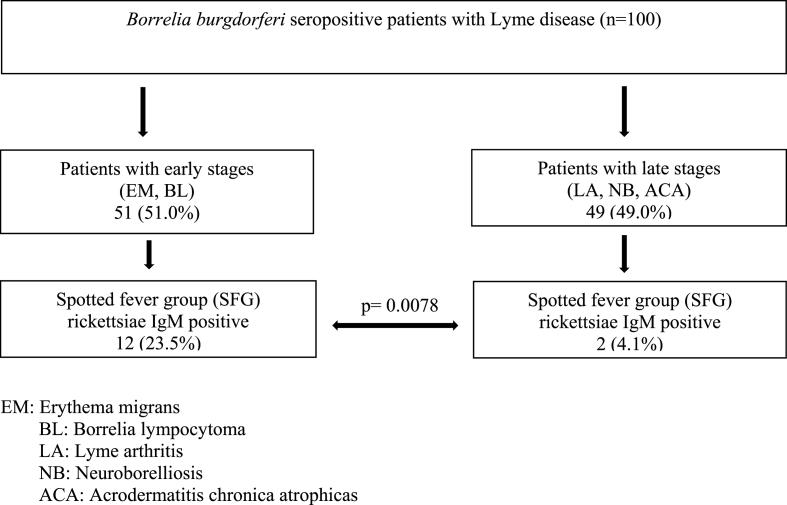
Seroprevalence of IgM antibodies against spotted fever group (SFG) rickettsiae in *Borrelia burgdorferi* seropositive patients with Lyme disease.

IgM antibodies were detected in 12 out of 51 (23.5%; 95% CI, 0.1387 to 0.3690) patients with early stages of Lyme borreliosis compared to 2 out of 49 (4.1%; 95% CI, 0.0035 to 0.1449) patients with late stages Lyme disease (p ​= ​0.0078; Fig. 2).

**Fig. 3:**
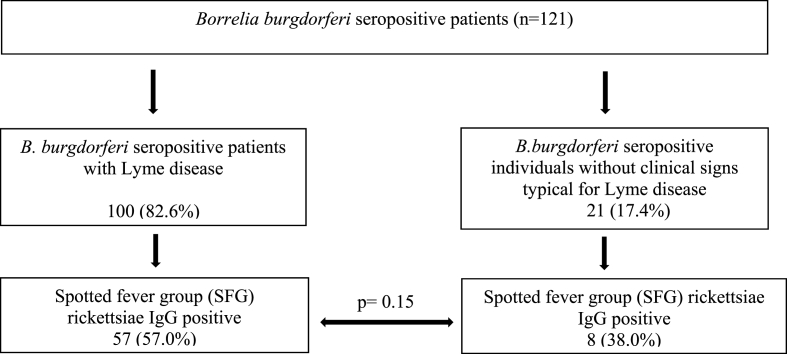
IgG antibodies against spotted fever group (SFG) rickettsiae in *B.burgdorferi* seropositive patients with Lyme disease and in individuals seropositive for *B. burgdorferi*, but without clinical signs typical for Lyme disease.

Comparable seroprevalence of IgG antibodies against SFG rickettsiae was observed in 27 out of 51 (52.9%; 95% CI, 0.3952 to 0.6595) patients with early stages and in 30 out of 49 (61.2%; 95% CI, 0.4722 to 0.7359) patients with late stages of Lyme disease (p ​= ​0.43). Similar IgG prevalence against SFG rickettsiae was found in 57 out of 100 (57%; 95% CI, 0.4721 to 0.6627) patients with Lyme disease compared to 8 out of 21 (38%; 95% CI, 0.2068 to 0.592) *B. burgdorferi* seropositive patients without typical clinical signs of Lyme disease (p ​= ​0.15; Fig. 3).

Significantly higher IgG antibody titers against SFG rickettsiae were found in patients with late-stage compared to patients with early-stage Lyme disease (mean titer 1:261 and 1:129, respectively; p ​= ​0.038). This difference was even more pronounced in patients with acrodermatitis chronica atrophicans compared to patients with early stage of Lyme disease (mean titer 1:337 and 1:129, respectively; p ​= ​0.009).

**Fig. 4:**
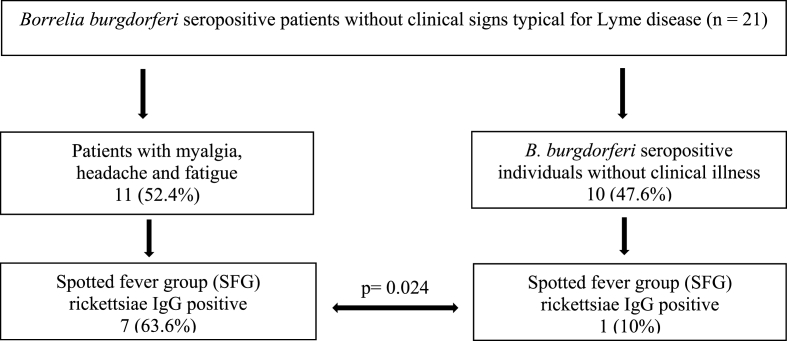
IgG antibodies against spotted fever group (SFG) rickettsiae in *B. burgdorferi* seropositive patients presenting with myalgia, headache and fatigue in comparison to *B. burgdorferi* seropositive individuals without clinical illness.

In patients presenting with fatigue, headache and myalgia, the prevalence of IgG antibodies against SFG rickettsiae was significantly higher (7 out of 11; 63.6%; 95% CI, 0.3519 to 0.8502) than in *B. burgdorferi* seropositive individuals without clinical illness (1 out of 10; 10%; 95% CI, <0.0001 to 0.4260; p ​= ​0.024; Fig. 4; Table 1, Table 2).

**Table 1:** IgG and IgM antibodies against spotted fever group (SFG) rickettsiae in Borrelia burgdorferi sensu lato seropositive patients presenting with myalgia, headache and fatigue.

Serum No.	Sex	Age	SFG rickettsiae IgG Antibody titer	SFG rickettsiae IgM Antibody titer
01-1844-4462	m	74	256	<8
01-1927-4978	f	21	<8	<8
01-1851-3109	f	66	128	<8
01-1917-3509	m	39	<8	<8
01-1850-1866	m	56	512	<8
01-1850-4977	m	77	64	8
01-1926-0711	f	49	128	128
01-1927-4322	f	52	<8	<8
01-1919-1867	f	61	128	8
01-1926-1644	m	55	128	<8
01-1847-3337	f	53	16	32

**Table 2:** IgG and IgM antibodies against spotted fever group (SFG) rickettsiae in Borrelia burgdorferi sensu lato seropositive individuals without clinical illness.

Serum No.	Sex	Age	SFG rickettsiae IgG Antibody titer	SFG rickettsiae IgM Antibody titer
01-1927-4317	f	42	16	<8
01-1927-0198	m	61	64	<8
01-1851-1032	m	52	32	<8
01-1927-4321	f	51	<8	<8
01-1943-4017	m	50	<8	<8
01-1845-5913	m	36	<8	8
01-1916-3180	m	49	<8	<8
01-1927-1547	f	73	<8	<8
01-1851-1030	f	66	16	<8
01-1927-4979	m	14	<8	<8

*A. phagocytophilum* IgG was detected in 12 out of 121 (9.9%; 95% CI, 0.0563 to 0.1667) and IgG antibodies to SFG rickettsiae in 65 out of 121 (53.7%; 95% CI, 0.4486 to 0.6235) individuals positive for *B. burgdorferi* antibodies (p ​< ​0.0001).

IgG antibodies solely to SFG rickettsiae were observed in 58 out of 121 (47.9%; 95% CI, 0.3923 to 0.5676), IgG antibodies to both SFG rickettsiae and *A. phagocytophilum* in 7 out of 121 (5.8%; 95% CI, 0.0263 to 0.1166) and IgG antibodies solely to *A. phagocytophilum* were detected in 5 out of 121 (4.1%; 95% CI, 0.0153 to 0.0956) individuals positive for *B. burgdorferi*.

In the control group no IgG antibodies to *A. phagocytophilum* were encountered. No *A. phagocytophilum* IgM antibodies were found in patients. One untreated subject in the control group showed IgM antibodies against *A. phagocytophilum* with a titer of 1024. In the follow-up testing IgM antibodies remained at the same level and no IgG seroconversion was observed.

## 4 Discussion

This retrospective serological study shows that not only *B. burgdorferi*, but also SFG rickettsiae and *A. phagocytophilum* are transmitted to humans during a tick bite in Switzerland. A significantly higher seroprevalence of IgG antibodies to SFG rickettsiae compared to *A. phagocytophilum* was observed. This difference can be explained by the fact that ticks in Switzerland are more frequently infected with SFG rickettsiae than with Anaplasma [12].

Antibodies to SFG rickettsiae and *A. phagocytophilum* were detected significantly more frequently in *B. burgdorferi* seropositive patients compared to the control group. *B. burgdorferi* seropositivity can be assumed as surrogate marker for a tick bite. This indicates that transmission of SFG rickettsiae and *A. phagocytophilum* is associated with tick bites and the majority of SFG rickettsiae positive patients are seropositive for *B. burgdorferi* as well.

A significantly higher prevalence of IgM antibodies against SFG rickettsiae was found in patients with early stage compared to late-stage Lyme borreliosis. This finding suggests a co-infection caused by a simultaneous transmission of *B. burgdorferi* and SFG rickettsiae during a tick bite. As expected, IgM antibodies to SFG rickettsiae decrease in titer after an acute infection and are no more detectable in patients with late Lyme disease.

While IgG seroprevalence to SFG rickettsiae showed no significant difference between early and late -stage Lyme disease patients, IgG titers were significantly higher in patients with late-stage Lyme disease and even more so in patients presenting with acrodermatitis chronica atrophicans. This finding suggests longer SFG rickettsiae persistence in these patients, whereas after a resolved infection, a titer decrease would be expected. One of the strategies that rickettsiae use to survive inside infected cells involves the inhibition of endothelial apoptosis [[23], [24], [25]].

A unique and salient feature of rickettsiae is tropism for the microvascular endothelium, resulting in disseminated inflammation, loss of barrier function, and altered vascular permeability - collectively referred to as rickettsial vasculitis. The characteristic histopathologic lesion is vasculitis with vascular and perivascular infiltration of predominantly CD4 and CD8 T lymphocytes and macrophages [23,26].

Especially early inflammatory stages of acrodermatitis chronica atrophicans show vasculitis and perivasculitis as also seen in rickettsial infections. Of particular interest is that ACA is much more common in Europe and less common in the United States. It may be that these could be increased in Europe by SFG rickettsiae [[27], [28], [29]].

Persistent infection with SFG rickettsiae could therefore be a coincidence or a potential co-factor in the pathogenesis of acrodermatitis chronica atrophicans. Further studies are needed to investigate this hypothesis.

Antibodies to SFG rickettsiae and *A. phagocytophilum* were detected with comparable frequency in samples of patients with and without clinical Lyme disease. This suggests that *B. burgdorferi* seropositivity, irrespectively of clinical presentation after a tick bite, can be used as a surrogate-marker for a possible co-transmission of SFG rickettsiae and *A. phagocytophilum*.

IgG antibodies to SFG rickettsiae were detected significantly more frequently in *B. burgdorferi* seropositive patients presenting with myalgia, headache, and fatigue compared to *B. burgdorferi* seropositive individuals without clinical disease. Symptoms such as headache, myalgia and fatigue are per se extremely common, but in the context of a tick-bite and positive SFG rickettsiae and/or *A. phagocytophilum* serology (especially in case of seroconversion or a fourfold titer increase) such symptoms have to be more carefully analysed, respecting other factors, such as living in a known endemic area for SFG rickettsiae and anaplasma. These microorganisms may pose a threat for populations exposed to *Ixodes ricinus* ticks. Special attention has to be paid if headache, myalgia and fatigue continue to persist after successful penicillin treatment of an early Lyme disease, because of penicillin resistance of SFG rickettsiae and *A. phagocytophilum*.

Based on our findings we can hypothesize that the combination of headache, myalgia and fatigue in individuals with high titers of IgG antibodies against SFG rickettsiae after a tick bite may be indicative of an infection with SFG rickettsiae [17,25]. To confirm our observations, further studies searching for seroconversion to SFG rickettsiae associated with the above-mentioned clinical symptoms are necessary. If patients are treated for Lyme disease with penicillin antibiotics, an infection with SFG rickettsiae and *A. phagocytophilum* should be ruled out because of resistance of these organisms to penicillins.

In patients with positive serology for SFG rickettsiae and/or *A. phagocytphilum* (especially in case of seroconversion or fourfold titer increase) associated with tick bite and clinical symptoms not consistent with Lyme disease, an appropriate therapy could be considered.

In the studied area roughly 50% of ticks harbour *B. burgdorferi* [9]. In addition, it is assumed that 0.3–1.4% of tick bites by *B. burgdorferi* infected ticks result in clinically manifest disease [30]. According to this estimation, our 100 patients with Lyme disease represent another 14,000–66,000 tick bites. After a tick bite, seroconversion is to be expected in 3–6% of those affected. This implies that our 21 ​B. burgdorferi seropositive individuals without Lyme disease represent further 700–1400 tick bites. Considering these data, we can consider that our patient population represents a large proportion of the population exposed to ticks.

Our study has certain limitations as follows.

The control and study groups were selected based on *B. burgdorferi* seropositivity as a surrogate marker for a tick bite, which is the decisive event for the transmission of tick-borne microorganisms. The selection of the control group was not simple because tick bites often go unnoticed [31]. Unfortunately, we were not able to exclude patients with an unnoticed tick bite by SFG rickettsiae and/or *A. phagocytophilum* positive ticks which are negative for *Borrelia burgdorferi* (unnoticed tick bite and negative surrogate marker). Such individuals would be attributed to a control group mistakenly. In our study only one patient would potentially belong to that group. This participant of the control group, without *B. burgdorferi* antibodies and without known tick bite showed IgG antibodies against SFG rickettsiae. Possible explanation of this unexpected seropositivity is an unnoticed tick bite by a tick carrying SFG rickettsiae and no *B. burgdorferi*. In a previous study in Sihlwald, ticks carrying SGF rickettsiae without *B.burgdorferi* have been observed [9]. One other participant of the control group had IgM and no IgG antibodies against *A. phagocytophilum*. He denied any known tick bite and had no symptoms suggestive for human anaplasmosis (or consistent with human anaplasmosis) and B. burgdorferi serology was negative. He did not get any antibacterial treatment. In a follow-up study, performed 11 months later, IgG antibodies to *B. burgdorferi* and *A. phagocytophilum* remained nondetectable and IgM antibodies were still present at the same titer. This constellation of serological results can be interpreted as false or non-specifically reactive for *A. phagocytophilum*.

Beside the *B. burgdorferi* seropositivity, the size, age and gender composition of each group were strongly influenced by the informed consent process and individual decision of each person to participate in the study (not all preselected and invited individuals participated in the study). Therefore, our data do not allow to comment on a potential effect of age and gender on seropositivity, and SFG rickettsiae antibody-titer. However, it seems unlikely that age or gender alone would explain seronegativity of all individuals but one in the control group.

In addition, our study did not include individuals with known tick bite and negative Borrelia serology, who have been bitten by SFG rickettsia and/or Anaplasma positive, but Borrelia negative ticks. In such individuals the duration of a tick bite would be another pitfall, because a short tick sucking time could significantly influence the probability of *B. burgdorferi*, SFG rickettsiae and *A. phagocytophilum* transmission.

One other study limitation was testing of a single patient specimen. Therefore, it was not possible to detect any seroconversion or antibody titer increase, consistent with an acute infection.

The main characteristic of our patient group and control group was that they live in the same area endemic for *B.burgdorferi*, SFG rickettsiae, and *A.phagocytophilum*. We do not have data from the subjects in the patient group regarding previous travel to SFG endemic areas.

For the detection of SFG rickettsiae, a group-specific *R. ricketsii* lipopolysaccharide antigen was used. Unfortunately, species-specific diagnostic tests are currently not commercially available. The use of the specific OmpA or OmpB antigens of *R. helvetica*, *R. monacensis*, and *R. slovaca* would possibly allow species-specific diagnosis of rickettsial infections. Such specific antigens are required for the extension of this study. They could result with higher sensitivity, higher antibody titers and possibly better correlation between serology and clinical presentation.

## 5 Conclusion

Our results suggest that tickborne infections with SFG rickettsiae and *A. phagocytophilum* as well as associated human disease are more widespread in Switzerland than previously considered. Despite high prevalence of SFG rickettsiae or *A. phagocytophilum* in ticks and a seroprevalence of more than 50% in individuals after a tick bite, human infections are rarely reported. Due to the lack of a typical clinical presentation infections with SFG rickettsiae and *A. phagocytophilum* are often not suspected, therefore these co-infections are often underdiagnosed and not specifically treated.

In patients with myalgia, headache, and persistent fatigue after a tick bite, infection with SFG rickettsiae should be suspected, tested, and possibly treated, unless they have already received adequate therapy. In patients with early Lyme disease, in whom antibody testing for SFG rickettsiae and *A. phagocytophilum* was not performed, tetracyline (specifically doxycycline) should be considered as first-line therapy after a tick bite to treat possible infection with SFG rickettsiae and *A. phagocyophilum* [[32], [33], [34]].

If antibiotic therapy with penicillins is preferred because of better tolerability, during pregnancy or in children, testing for antibodies to SFG rickettsiae and *A. phagocytphilum* should be performed after a tick bite. Species-specific antigens are needed for serotyping and differentiating SFG rickettsiae at the species level.

Further studies on the prevalence and clinical significance of SFG rickettsiae and *A. phagocytophilum* should be conducted. A possible role of SFG rickettsiae as a co-factor in late stages of Lyme disease such as acrodermatitis chronica atrophicans needs to be further investigated. Prospective studies should be performed to elucidate the possible causative role of SFG rickettsiae for myalgia, headache and long-lasting fatigue after a tick bite.

## Consent for publication

It is to certify that all authors have seen and approved the final version of the manuscript submitted. They warrant that the article is the authors' original work, hasn't received prior approval for publication and isn't under consideration for publication elsewhere.

## Transparency declaration

The authors declare no conflict of interest. This research was funded by Analytica Medizinische Laboratorien AG, Zurich, Switzerland.

## CRediT authorship contribution statement

**Leonie Kosak:** Conceptualization, Funding acquisition, Formal analysis, Data curation. **Norbert Satz:** Funding acquisition, Formal analysis, Data curation. **Markus Jutzi:** Funding acquisition, Formal analysis, Data curation. **Marinko Dobec:** Conceptualization, Funding acquisition, Formal analysis, Data curation. **Patricia Schlagenhauf:** Funding acquisition, Formal analysis, Data curation.
